# ^18^F-FDG-PET Can Predict Microvessel Density in Head and Neck Squamous Cell Carcinoma

**DOI:** 10.3390/cancers11040543

**Published:** 2019-04-15

**Authors:** Alexey Surov, Hans Jonas Meyer, Anne-Kathrin Höhn, Andreas Wienke, Osama Sabri, Sandra Purz

**Affiliations:** 1 Department of Diagnostic and Interventional Radiology, University Hospital of Leipzig, Liebigstrasse 20, 04103 Leipzig, Germany; hans-jonas.meyer@medizin.uni-leipzig.de; 2 Department of Pathology, University Hospital of Leipzig, Liebigstrasse 20, 04103 Leipzig, Germany; Annekathrin.hoehn@medizin.uni-leipzig.de; 3 Institute of Medical Epidemiology, Biostatistics, and Informatics, Martin-Luther-University Halle-Wittenberg, Magdeburger Str. 8, 06097 Halle, Germany; andreas.wienke@uk-halle.de; 4 Department of Nuclear Medicine, University Hospital of Leipzig, Liebigstrasse 18, 04103 Leipzig, Germany; Osama.Sabri@medizin.uni-leipzig.de (O.S.); sandra.purz@medizin.uni-leipzig.de (S.P.)

**Keywords:** positron emission tomography, head and neck neoplasms, neovascularization, pathologic

## Abstract

*Aim*: Positron emission tomography (PET) with ^18^F-fluordeoxyglucose (^18^F-FDG) plays an essential role in the staging and tumor monitoring of head and neck squamous cell carcinoma (HNSCC). Microvessel density (MVD) is one of the clinically important histopathological features in HNSCC. The purpose of this study was to analyze possible associations between ^18^F-FDG-PET findings and MVD parameters in HNSCC. *Materials and Methods*: Overall, 22 patients with a mean age of 55.2 ± 11.0 and with different HNSCC were acquired. In all cases, whole-body ^18^F-FDG-PET was performed. For each tumor, the maximum and mean standardized uptake values (SUV_max_; SUV_mean_) were determined. The MVD, including stained vessel area and total number of vessels, was estimated on CD105 stained specimens. All specimens were digitalized and analyzed by using ImageJ software 1.48v. Spearman’s correlation coefficient (*r*) was used to analyze associations between investigated parameters. *p*-values of <0.05 were taken to indicate statistical significance. *Results:* SUV_max_ correlated with vessel area (*r* = 0.532, *p* = 0.011) and vessel count (*r* = 0.434, *p* = 0.043). Receiver operating characteristic analysis identified a threshold SUV_max_ of 15 to predict tumors with high MVD with a sensitivity of 72.7% and specificity of 81.8%, with an area under the curve of 82.6%. *Conclusion:* ^8^F-FDG-PET parameters correlate statistically significantly with MVD in HNSCC. SUV_max_ may be used for discrimination of tumors with high tumor-related MVD.

## 1. Introduction

Radiological imaging, especially positron emission tomography (PET) with ^18^F-fluorodeoxyglucose (^18^F-FDG), plays an essential role in characterization head and neck squamous cell carcinoma (HNSCC). ^18^F-FDG-PET is increasingly used for the staging and treatment monitoring of HNSCC [1,2,3]. As reported previously, metabolic tumor activity measured via PET parameters such as maximum or mean standardized uptake values (SUV_max_ or SUV_mean_) correlates well with tumor stage and grade [2,3]. Advanced T stage tumors show higher PET parameters like SUV_max_ and SUV_mean_ in comparison to T1/T2 tumors [2,3]. Similarly, poorly differentiated (G3) tumors have higher SUV_max_ than do low-grade (G1 or G2) lesions [3]. Furthermore, the metabolic tumor burden is associated with the clinical outcome of HNSCC: patients with high metabolic tumor burden have been associated with higher distant metastasis rates, translating into worse survival [4,5]. In addition, ^18^F-FDG-PET can also predict treatment success in HNSCC. It has been shown that SUV values can be used as biomarkers in predicting the therapy response in HNSCC [6,7]. Kitagawa et al. reported that the SUV of pre-treatment ^18^F-FDG-PET is useful in predicting the response to treatment, and post-treatment ^18^F-FDG-PET is valuable in predicting residual viable tumors. It was also mentioned that a lower SUV (<4) of post-treatment ^18^F-FDG-PET was significantly correlated with good histological results after therapy [6].

Some reports indicated that ^18^F-FDG-PET can reflect several clinically relevant histopathological features in HNSCC. So far, Jacob et al. found that SUV_max_ correlated statistically significantly with the proliferation index KI 67 (*r* = 0.78) and proliferating cell nuclear antigen (*r* = 0.66) [8]. Grönroos et al. showed that SUV_max_ tended to correlate with the expression of tumor suppressor protein p53 (*p* = 0.47, *p* = 0.078) [9]. Furthermore, SUV_max_ also correlated well with the expression of hypoxia-inducible factor HIF-1α [10]. It has also been shown that p16-positive tumors had lower SUV_max_ in comparison to p16-negative carcinomas [11,12].

According to the literature, microvessel density (MVD) also plays a significant role in HNSCC [13]. For example, MVD estimated from CD105 immunoexpression predicts a poor outcome in oral squamous cell carcinoma [13,14]. Like VEGF (vascular endothelial growth factor), CD105 (endoglin) is a hypoxia-inducible transmembrane glycoprotein, and its expression is up-regulated in actively proliferating endothelial cells. Endoglin has been described as a marker for tumor-related angiogenesis and neovascularization with potential in tumor diagnosis, prognosis, and therapy [15]. Xia et al. found that MVD can predict lymph node metastases and prognosis in HNSCC [16]. We assume that the parameters of ^18^F-FDG-PET might also reflect MVD in HNSCC. However, no previous study has investigated the relationships between ^18^F-FDG-PET and tumor MVD in HNSCC.

Therefore, the purpose of the present study was to analyze possible associations between ^18^F-FDG-PET parameters and MVD in HNSCC.

## 2. Methods

This prospective study was approved by the institutional review board (Ethics Committee of the University of Leipzig, study codes 180-2007, 201-10-12072010, and 341-15-05102015). All methods were performed in accordance with the relevant guidelines and regulations. All patients gave their written informed consent.

### 2.1. Patients

For this study, patients with histologically proven HNSCC and available histopathological specimens and who underwent ^18^F-FDG-PET/CT examinations at our institution were selected. Overall, there were 22 patients, 6 (26.1 %) women and 16 (73.9 %) men, with a mean age of 55.2 ± 11.0 years, age range of 24–77 years, and different HNSCC. Low-grade (G1/2) tumors were diagnosed in 10 cases (45.5%) and high-grade (G3) tumors in the remaining 12 (54.5%) patients.

### 2.2. Imaging

#### ^18^F-FDG-PET/CT

In all 22 patients, an ^18^F-FDG-PET/CT (Siemens Biograph 16, Siemens Medical Solutions, Erlangen, Germany) was performed from the skull to the upper thigh after a fasting period of at least 6 h. Application of ^18^F-FDG was performed intravenously with a body-weight-adapted dose (4 MBq/kg, range: 168–427 MBq, mean ± SD: 281 ± 62.2 MBq). PET/CT image acquisition started on average 76 min (range 60–90 min) after ^18^F-FDG application. Low-dose CT was used for attenuation correction of the PET data.

**Figure 1:**
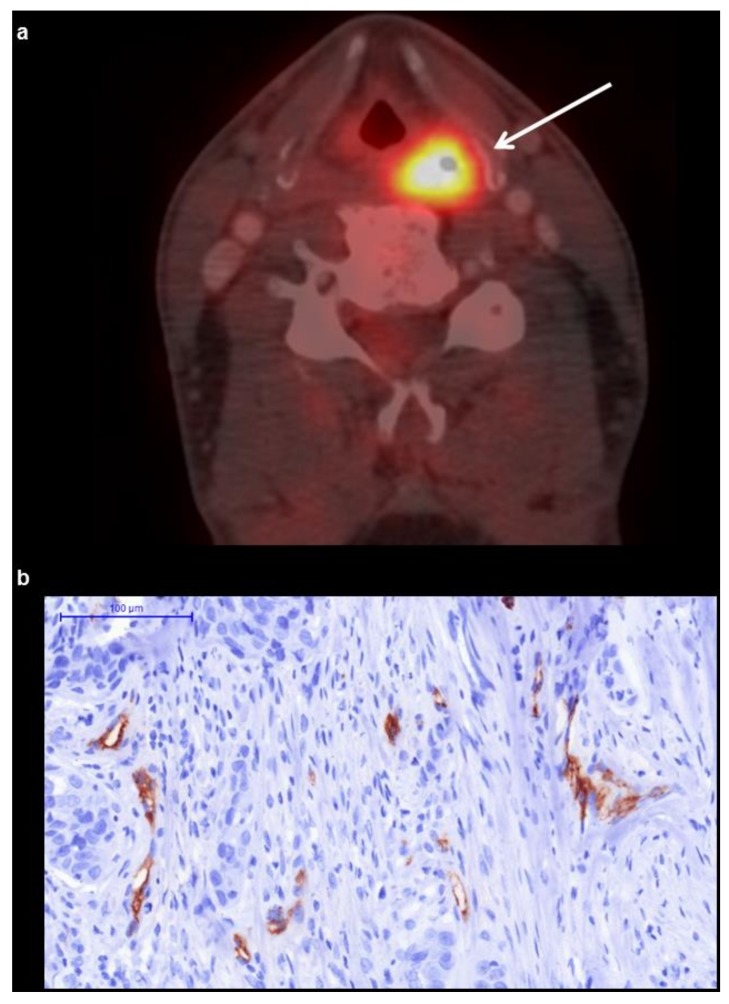
(**a**) ^18^F-fluordeoxyglucose (^18^F-FDG)-PET/CT shows a metabolically active hypopharyngeal lesion. The acquired ^18^F-FDG-PET parameters of the lesion are as follows: maximum and mean standardized uptake values SUV_max_ = 22.07 and SUV_mean_ = 13.92. magnification: 200×. (**b**) Histopathological findings (CD 105 stained specimen). Vessel area is 1.2%, vessel count is 11.

The acquired PET/CT datasets were evaluated by a board-certified nuclear medicine practitioner and a board-certified radiologist with substantial PET/CT experience in oncological image interpretation. PET/CT image analysis was performed on a dedicated workstation at Hermes Medical Solutions, Sweden. For each tumor, the maximum and mean SUV (SUV_max_; SUV_mean_) were determined from PET images (Figure 1). Prior to this, the tumor margins of the HNSCC were identified on CT images and fused PET/CT images, and a polygonal volume of interest (VOI) that include the entire lesion in the axial, sagittal, and coronal planes was placed in the PET dataset (SUV_max_ threshold 40%).

### 2.3. Microvessel Density

In all cases, the diagnosis was confirmed histopathologically by tumor biopsy before any form of treatment. For the present study, the biopsy specimens were deparaffinized, rehydrated, and cut into 5 μm slices. The specimens were stained with CD 105 antigen (Abcamplc, 330 Cambridge Science Park, Cambridge, CB4 0FL, UK). Furthermore, all stained specimens were digitalized by using a Pannoramic microscope scanner (Pannoramic SCAN, 3DHISTECH Ltd., Budapest, Hungary) with Carl Zeiss objectives up to 41× bright field magnification by default. In the used bottom-up approach, the whole sample was acquired at high resolution. The digital slides (magnification of 200×) were evaluated using Pannoramic Viewer 1.15.4 (open source software, 3D HISTECH Ltd., Budapest, Hungary).

Thereafter, the digitalized histopathological images were analyzed using ImageJ software 1.48v (National Institutes of Health Image program) with a Windows system [17,18]. The microvessel density included the following parameters: stained vessel area (vessel area, % per high-power field), calculated as the CD105 positive area divided by the total area of the analyzed histological specimens, and the total number of vessels (vessel count) according to Weidner et al. [19].

### 2.4. Statistical Analysis

Statistical analysis was performed using the SPSS package (IBM SPSS Statistics for Windows, version 22.0, Armonk, NY, USA: IBM corporation). The collected data were evaluated by means of descriptive statistics. The statistical data included means and medians with corresponding standard deviations and ranges of the acquired ^18^F-FDG-PET and histopathological parameters.

Spearman’s correlation coefficient (*r*) was used to analyze associations between the investigated variables. *p*-values of <0.05 were taken to indicate statistical significance. Furthermore, the sensitivity, specificity, negative and positive predictive values, accuracy, and area under the receiver operating characteristic curve (AUC) value were calculated for the diagnostic procedures. Thresholds were chosen to maximize the Youden index.

## 3. Results

Information regarding tumor localization, stage, and grade of the enrolled patients is given in Table 1. Table 2 shows a complete overview of the acquired ^18^F-FDG-PET and histopathological parameters including mean values, standard deviations, and ranges.

**Figure 2:**
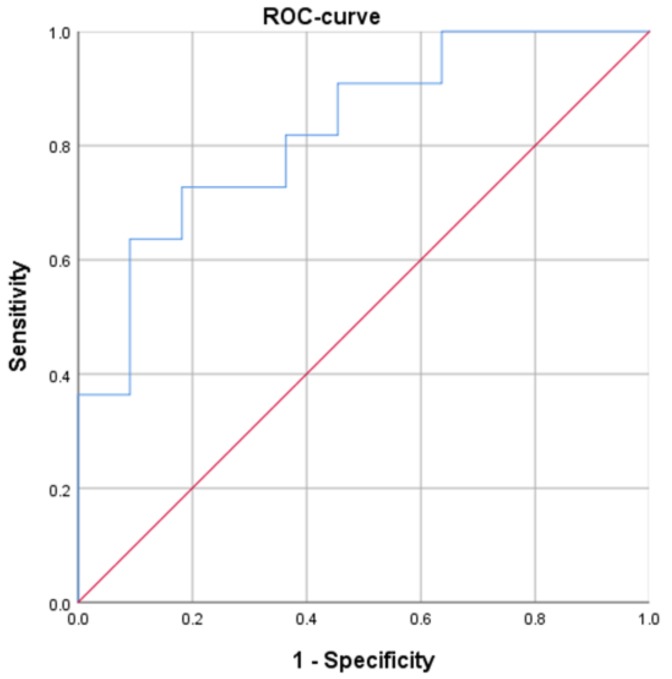
The receiver operating characteristic (ROC) curve using SUV_max_ for distinguishing tumors with high microvessel density from lesions with low microvascularization. The optimal threshold SUV_max_ is 15, resulting in a sensitivity of 70.0% and a specificity of 75.0%. The area under the curve is 82.6%.

SUV_max_ correlated statistically significantly with vessel area (*r* = 0.532, *p* = 0.011) and vessel count (*r* = 0.434, *p* = 0.043). SUV_mean_ also correlated statistically significantly with vessel area (*r* = 0.465, *p* = 0.029). In the next step, receiver operating characteristic (ROC) analysis was performed to predict tumors with high microvessel density (vessel area > 1.76% as a result of median split) using SUV_max_. The Youden index identified a threshold SUV_max_ of 15 with a sensitivity of 72.7% and specificity of 81.8% (Figure 2). The positive predictive value was 80%, the negative predictive value was 75%, and the accuracy was 77.3%. The area under the curve was 82.6%.

## 4. Discussion

The present study identified significant associations between parameters of ^18^F-FDG-PET and MVD in HNSCC. According to the literature, MVD is a very important histopathological feature in different malignancies. There are different immunohistochemical markers for the estimation of MVD. Some authors used pan-endothelial markers, namely, CD34, CD31, and von Willebrand factor [19,20,21]. However, it is well known that these markers have low sensitivity and specificity and are not always expressed in all intratumoral vessels [21]. In contrast to the non-specific pan-endothelial markers, CD105 or endoglin is upregulated in angiogenic vessels and accumulates preferentially in tumors [22,23]. Therefore, CD105 is a marker of tumor-related MVD.

Previously, numerous studies showed that CD105 correlated with tumor aggressiveness in lung cancer [24,25], breast carcinomas [26], colonic cancer [27], and endometrial carcinoma [28]. MVD is also one of the clinically relevant features in HNSCC [29,30,31,32]. According to the literature, tumor size/T stage correlated directly with the MVD/CD 105 ratio in oral cancers [31]. Furthermore, some reports indicated that high MVD/CD105 values were associated with the presence of lymph node metastasis in HNSCC [29,32,33]. Finally, high MVD/expression of CD105 was also associated with recurrence of disease or occurrence of distant metastasis and poorer 5-year survival [33,34,35,36,37]. Therefore, MVD can be applied as an important independent prognostic factor in HNSCC.

The possibility to predict MVD from imaging is very important. Previously, relationships between ^18^F-FDG-PET findings and MVD were analyzed in several malignancies. Remarkably, different correlation coefficients were observed between the investigated parameters. Furthermore, different markers for MVD were used. Han et al. used CD34 marker and did not observe statistically significant correlations between SUV_max_ and MVD in lung cancer [38]. However, Xing et al. also estimated MVD from CD 34 stained specimens and reported a very strong correlation between tumor MVD and SUV in lung cancer (*r* = 0.915, *p* < 0.01) [39]. In esophageal cancer, no significant correlations were detected between PET parameters and MVD measured from CD31 stained specimens [40]. Only few studies have analyzed the associations between PET and tumor-related MVD based on CD 105 expression. In the study by Groves et al., SUV_max_ correlated statistically significantly with MVD (*r* = 0.6, *p* = 0.005) in breast cancer [41]. Cochet et al. also investigated patients with breast cancer but could not identify statistically significant correlations between SUV_max_ and the expression of CD105 or CD34 [42]. However, interestingly, in the same study, SUV_max_ was associated with expression of CD105 in a non-triple-negative tumor subgroup (*r* = 0.5, *p* = 0.005) [42]. Finally, in colorectal cancer, no significant correlations between metabolic parameters (SUV_max_ or SUV_mean_) and CD 105 expression were found [43].

In HNSCC, there have been no previous studies about associations between ^18^F-FDG-PET and tumor-related MVD. The present study showed that SUV_max_ may predict MVD in HNSCC. Moreover, SUV_max_ may be used for discrimination of tumors with higher expression of CD 105, i.e., high-risk lesions. This finding is very important. It may help us to identify patients suitable for targeted therapy with anti-angiogenetic antibodies (e.g., anti-endoglin) in an oncological therapeutic setting. However, in agreement with the results by Groves et al. [41], the identified correlations were moderate. Also, the calculated negative and positive predictive values and the area under the curve and accuracy were relatively low. These facts limit the use of the present data to make a clinical decision.

Our study also identified another interesting aspect. The calculated correlation coefficients between SUV_max_ and MVD are comparable with those for specific perfusion parameters. In fact, as reported previously, one of the parameters of dynamic contrast-enhanced MRI, namely Kep, correlated with vessel area (*r* = 0.51, *p* = 0.041) in HNSCC [44]. Blood volume, a parameter of CT perfusion, correlated with vessel count (*r* = 0.59, *p* = 0.035) [45]. Furthermore, in the present study, we analyzed important tumor-related MVD estimated from CD 105 expression. Previous reports, however, investigated MVD using the expression of CD 31 or CD 34 [29,30]. According to the literature, these markers are not tumor-specific and do not play a clinical role in HNSCC [14,27]. The present study is limited due to a small number of patients. Clearly, further investigations with more cases are needed to verify our results.

## 5. Conclusions

^18^F-FDG-PET parameters correlated statistically significantly with MVD in HNSCC. SUV_max_ may be used for discrimination of tumors with high tumor-related MVD.

## Figures and Tables

**Table 1:** Clinical characteristics of the patients enrolled in the study.

No.	Sex	Age	Tumor Site	T Stage	N Stage	M Stage	Grading
1	female	33	Oral cavity	3	0	0	2
2	male	62	Larynx	3	3	0	3
3	male	55	Oropharynx	3	2	0	3
4	male	56	Hypopharynx	3	1	0	3
5	female	58	Oropharynx	1	2	0	3
6	male	24	Oral cavity	4	2	0	2
7	male	64	Oral cavity	2	1	0	3
8	male	57	Oropharynx	2	2	0	3
9	male	44	Larynx	4	0	0	3
10	female	77	Epipharynx	4	1	1	3
11	male	59	Oropharynx	3	1	0	2
12	male	53	Larynx	4	2	0	3
13	male	64	Hypopharynx	4	2	0	2
14	male	61	Oropharynx	4	2	0	2
15	male	58	Oropharynx	2	2	0	2
16	female	60	Oropharynx	4	2	0	4
17	male	55	Oropharynx	3	2	0	2
18	male	54	Oral cavity	4	2	0	2
19	female	65	Oropharynx	2	2	0	3
20	male	50	Oropharynx	2	2	0	3
21	male	48	Hypopharynx	2	2	0	2
22	female	58	Oral cavity	4	2	0	1

**Table 2:** Estimated ^18^F-FDG-PET and microvessel density (MVD) parameters of head and neck squamous cell carcinoma (HNSCC).

Parameters	M ± SD	Median	Range
SUV_max_	14.34 ± 5.05	14.79	5.9–24.1
SUV_mean_	8.40 ± 3.11	8.28	3.63–14.87
Vessel Area	1.97 ± 1.15	1.76	0.4–4.56
Vessel Count	11.64 ± 4.97	10	5–25

## Abbreviations

^18^F-FDG	^18^F-fluorodeoxyglucose
HNSCC	head and neck squamous cell carcinoma
MVD	micro vessel density
PET	positron emission tomography
ROC	receiver operating characteristic
SUV	standardized uptake values
